# The Need for Common Sense Extension

**Published:** 1919-06-14

**Authors:** 


### VACCINATION MEASURES.

### The Need for Common Sense Extension.

FOR some weeks past both the medical and lay Press has made occasional reference to the fresh incidence of smallpox in England, and now again, in a recent issue of the *Lancet*, attention is drawn to the subject by publication of several details of this matter during the present year. It is very obvious that our relative immunity from virulent outbreaks in the past has not only allowed our unvaccinated percentage of the population to increase alarmingly, but also to permit of considerable forgetfulness on the part of the laity in common-sense measures of self-protection, and, as seems more than probable, on the part of the professional authorities, in bringing the true requirements prominently and publicly to their notice.

Among the sixty instances recorded in England and Wales since the beginning of the year, one notes that besides being scattered over a number of districts in the London area, cases are recorded in localities as far apart as Liverpool, Hartlepool, Plymouth, Cardiff, and Bury St. Edmunds, and, although the local prevalence at present is nowhere sufficiently great to be called a true epidemic, yet the figures point only too clearly to the fact that the menace is by no means localised.

Referring to the origin of these cases, the *Lancet* explains that in half-a-dozen districts the infection appears to have been introduced by demobilised soldiers returning to this country *via* Taranto and and Havre. Considerations of the date of onset and incubation period point to the fact that infection occurred after arrival in Italy and before departure from France. Demobilisation centres were passed through while the men were incubating the disease, and the first symptoms did not become manifest until the cases had reached their homes. Similarly, a number of people have probably brought the disease from Portugal, for incubation takes longer than the period occupied by the relatively short voyage made.

Be the exact origin what it may, the fact remains that we have these cases among us, and the most surprising thing is that only now, after several cases have occurred among unvaccinated or inefficiently vaccinated workers in fever hospitals, are those concerned bestowing active attention upon the need for general re-vaccination. It must be remembered that persons actually employed in medical practice, health inspecting, and institutional work, are particularly liable to carry the disease further afield. Certainly we have up to now promptly hospitalised the cases as they occurred, but their menace, though removed from the public, is still indirectly present through the attendants. The duty of the latter, therefore, is to be absolutely certain of their own vaccination even before spreading advice to those under their care. One hears of a number of instances of infection among nurses, sanitary workers, and in one instance, of a medical man himself, and as the *Lancet* wisely advises—

> All sanitary officials should, of course, be vaccinated as soon as, or preferably before, smallpox appears in the district. In the case of those with families, each member of the family should be vaccinated or re-vaccinated, as the case may be. Ward-maids and porters at smallpox hospitals, and ambulance drivers and undertakers who may come in contact with smallpox should also be vaccinated; and, in view of the number of cases of the malady occurring in different parts of the country at present, vaccination or re-vaccination as a precautionary measure would be reasonable.

Not only reasonable, we think, but from many points of view a necessity of great urgency. It is of considerable importance that the more virulent strains of this infection should not again become endemic in the country, and whatever our means of controlling the number of actual cases, it is most necessary that the minimum opportunity of development in the unvaccinated people should be given to smallpox. One refers to the matter of virulence rather than actual prevalence, and from this view at least we suggest that a universal and organised campaign throughout the country to ensure revaccination of every person known to be in need of it. A few years ago a far less serious menace caused this to be done, partly officially, but largely voluntarily. The present is assuredly both a favourable and desirable moment to reinitiate such a protective movement.

